# Natural Selection on the Phase-Separation Properties of FUS during 160 My of Mammalian Evolution

**DOI:** 10.1093/molbev/msaa258

**Published:** 2020-10-06

**Authors:** Pouria Dasmeh, Andreas Wagner

**Affiliations:** 1 Institute for Evolutionary Biology and Environmental Studies, University of Zurich, Zurich, Switzerland; 2 Department of Chemistry and Chemical Biology, Harvard University, Cambridge, MA; 3 Swiss Institute of Bioinformatics (SIB), Lausanne, Switzerland

**Keywords:** FUS, prion-like domain, liquid–liquid phase separation

## Abstract

Protein phase separation can help explain the formation of many nonmembranous organelles. However, we know little about its ability to change in evolution. Here we studied the evolution of the mammalian RNA-binding protein Fused in Sarcoma (FUS), a protein whose prion-like domain (PLD) contributes to the formation of stress granules through liquid–liquid phase separation. Although the PLD evolves three times as rapidly as the remainder of FUS, it harbors absolutely conserved tyrosine residues that are crucial for phase separation. Ancestral reconstruction shows that the phosphorylation sites within the PLD are subject to stabilizing selection. They toggle among a small number of amino acid states. One exception to this pattern is primates, where the number of such phosphosites has increased through positive selection. In addition, we find frequent glutamine to proline changes that help maintain the unstructured state of FUS that is necessary for phase separation. Our work provides evidence that natural selection has stabilized the liquid forming potential of FUS and minimized the propensity of cytotoxic liquid-to-solid phase transitions during 160 My of mammalian evolution.

## Introduction

Protein phase separation is a thermodynamic process through which a well-mixed solution of proteins and DNA/RNA molecules demixes into two phases of high and low densities (Shin and Brangwynne 2017; Boeynaems et al. 2018). It has emerged as a biophysical mechanism to explain the formation of several membrane-less organelles and biomolecular condensates in living cells. Examples of these condensates include the nucleolus, which is involved in ribosome assembly (Mitrea et al. 2018), cytoplasmic granules that form when cells are stressed (Bergeron-Sandoval et al. 2016; Banani et al. 2017; Riback et al. 2017; Franzmann et al. 2018), and RNA processing bodies involved in mRNA metabolism and turnover (Kroschwald et al. 2015).

One mammalian protein whose phase separation is well-studied is the RNA-binding protein Fused in Sarcoma (FUS) (Vance et al. 2009; Mackenzie et al. 2010; Kapeli et al. 2016). FUS is predominantly a nuclear protein and regulates the mRNA life cycle at different stages, from pre-mRNA splicing to mRNA transport and translation. In addition, FUS directly interacts with Poly-ADP-ribose polymerase and mediates DNA damage response in the cell (Naumann et al. 2018). FUS self-assembles through the interaction of its N-terminal prion-like domain (PLD) and the arginine–glycine–glycine domains (Kato and McKnight 2017; Murray et al. 2017). FUS requires this self-assembly for its nuclear functions, such as binding to chromatin and recruitment to the sites of DNA damage (Mastrocola et al. 2013; Altmeyer et al. 2015).

FUS most likely behaves as a multivalent heteropolymer composed of simple sequence motifs, also called stickers (Wang et al. 2018). These motifs consist of arginine residues in the arginine–glycine–glycine domain, and of tyrosine residues in the low complexity PLD. Their interaction is the major driving force for the phase separation of FUS (Wang et al. 2018).

Sticker amino acids are interspersed with sequence segments called spacers. Spacers can modify the properties of polypeptide chains and regulate the formation kinetics or the material states of phase-separated condensates (Harmon et al. 2017; Choi et al. 2019). In addition to this polymerization behavior, the formation of amyloid-like cross-beta sheet structures might play a role in the self-assembly of FUS droplets. Within the PLD, a region of 100 amino acid residues is especially prone to form these labile beta-sheet structures (Murray et al. 2017). Phosphorylation of several serine and threonine residues within this region disrupts these aggregates and regulates FUS assembly in the cell (Monahan et al. 2017; Murray et al. 2017).

The liquid-like state of FUS is also stabilized by a diverse set of molecular interactions within the PLD (Murthy et al. 2019). This dynamic state is exquisitely sensitive to single-point mutations. In fact, missense mutations in FUS occur in patients with the neurodegenerative diseases amyotrophic lateral sclerosis (ALS) and frontotemporal lobar degeneration (Mackenzie et al. 2010). For instance, a single ALS-associated mutation, G156E, facilitates a liquid-to-solid phase transition of FUS into irreversible aggregates (Patel et al. 2015).

The importance of FUS in the life of cells, together with the sensitivity of FUS assemblies to point mutations, raises the possibility that natural selection must actively maintain the ability of FUS to form the liquid-droplet state. We thus hypothesized that evolution has preserved the phase-separation propensity of FUS, and avoids the pathological liquid-to-solid phase separation in FUS, just like it maintains folding stability and reduces misfolding in proteins with structured domains (Zhang and Yang 2015).

## Results

**Fig. 1.**
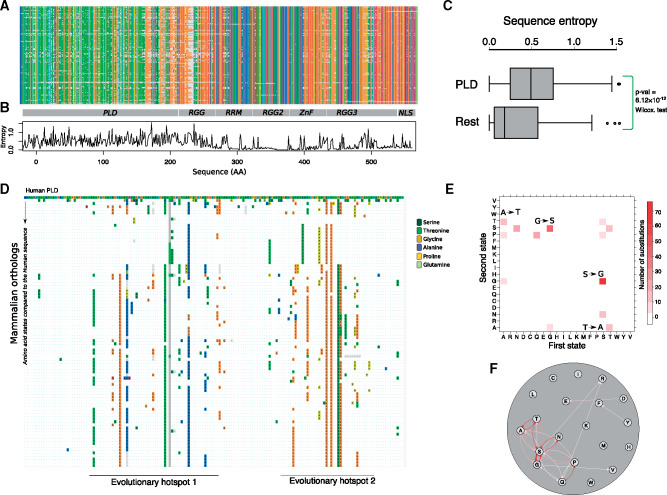
The PLD is the most variable domain of FUS in mammals. (*A*) Multiple sequence alignments of 105 mammalian FUS orthologs colored according to the CLUSTAL color scheme (Larsson 2014). (*B*) Shannon entropy of each amino acid site in the alignment. (*C*) Box plots comparing the sequence entropy of the amino acid sites in the PLD with the rest of the residues in FUS. (*D*) Substitution map of the PLD in mammals. The first row corresponds to the PLD sequence of human FUS color-coded in the CLUSTAL format. The following rows show the sequences of mammalian PLDs compared with the sequence of the human PLD in the first row. If, at any position, the amino acid is different from that of the human PLD, the new amino acid is shown by colored boxes. Identical amino acids are shown as blue dots. Amino acid substitutions involve changes to serine (forest green), threonine (green), glycine (orange), alanine (blue), proline (yellow), and glutamine (Emerald green). (*E*) Ancestral state mapping of substitutions in the evolution of PLD. Each box represents a substitution with its color saturation proportional to the number of such replacements. The first and second amino acids in a substitution are shown on the X and Y axes, respectively. Arrows highlight the especially frequent A to T, G to S, S to G, and T to A substitutions. (*F*) Ancestral state mapping from € represented as a directed graph. Each circle or node represents one amino acid, and substitutions are shown as edges that connect these nodes. The thickness of each edge corresponds to the number of substitutions between the two incident nodes. Substitutions with more than Ancestral state mapping ten occurrences are shown in red.

To identify the forces and mechanisms that govern the evolution of liquid–liquid phase separation, we studied how FUS evolved in mammals. We first identified 105 mammalian orthologs of FUS, aligned them (fig. 1*A*), and computed each residue’s sequence entropy, a widely used measure of sequence divergence (fig. 1*B*). The PLD, which is central for FUS phase separation, has the highest sequence entropy of all FUS domains, with a median ∼3-fold higher than that of the other FUS domains (fig. 1*C*; Wilcoxon rank-sum test, *P* = 6.12 × 10^−12^), and it shows that the PLD domain evolves much faster than the rest of FUS. Nonetheless, tyrosine residues within this domain are fully conserved. Since the interaction of tyrosine residues with arginines in the RNA-binding domain of FUS is essential for phase separation (Wang et al. 2018), this strong conservation might indicate that evolution has maintained the phase-separation propensity of FUS in mammals

Within the PLD, we observed two evolutionary hotspots, which are the regions S30 to S86, and A105 to Q147 (all site numbers and amino acids refer to human FUS). These regions are subject to multiple substitutions that involve the amino acids glycine, serine, alanine, threonine, asparagine, proline, and glutamine (fig. 1*D*). By reconstructing ancestral FUS proteins (supplementary tables S1 and S2, Supplementary Material online, see Materials and Methods for details), we found that changes where the PLD sites toggle forth and back between G and S (113 changes), as well as between A and T (34 changes), are especially prevalent (fig. 1*E and F*). Together, these changes account for ∼60% of all changes in the evolution of the PLD. In addition, we found 32 switches between serine and asparagine, and 20 switches between glutamine and proline in these evolutionary hotspots (supplementary tables S1 and S2, Supplementary Material online). To exclude the possibility that the high rates of substitutions between glycine and serine as well as alanine and threonine are caused by inaccurate ancestral reconstruction of the intrinsically disordered PLD, we repeated our analysis, but only counted substitutions in residues with a reconstruction accuracy of >90%. We found that these substitutions still account for ∼55% of all substitutions (supplementary fig. S4 and table S9, Supplementary Material online). We observed the same percentage when we used a substitution matrix developed to model the evolution of intrinsically disordered mammalian proteins (Szalkowski and Anisimova 2011) (supplementary fig. S5 and table S9, Supplementary Material online). In sum, the high rate of G to S and A to T substitution is an inherent feature of PLD evolution rather than an artifact of ancestral sequence reconstruction.

**Fig. 2.**
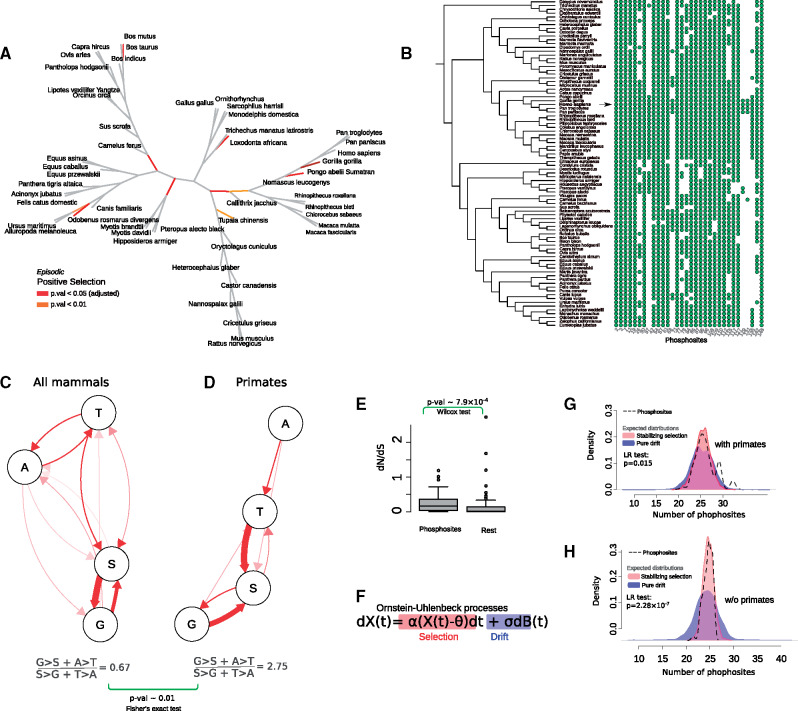
Positive selection in the evolution of phosphorylation sites. (*A*) Mammalian phylogenetic tree showing the branches under positive selection in the evolution of the PLD. Branches on which positive selection is detected with significance levels of *P* = 0.01 and *P* = 5 × 10^−4^ (adjusted *P* = 0.05, using Bonferroni correction [Bonferroni et al. 1936] with 97 branches), respectively, are colored in orange and red (*P* values corrected for multiple sampling). (*B*) Mammalian phylogenetic tree, along with a map indicating the presence (green circle) or absence (blank) of serines/threonines in the phosphorylation sites of human FUS (arrow). (*C*) Ancestral state mapping of substitutions involving A, T, S, G for the whole phylogenetic tree. (*D*) Ancestral state mapping of substitutions involving A, T, S, G for primates. The *P* value is calculated from Fisher’s exact test (supplementary information, Supplementary Material online). (*E*) Boxplots comparing the rate of evolution (dN/dS) of phosphosites with the rest of the PLD residues. (*F*) The general stochastic equation of an OU process with drift and selection components, highlighted in blue and red, respectively. (*G* and *H*) Probability density of the number of phosphosites (dashed line) compared with the probability density of the evolution of phosphosites under drift (blue) and stabilizing selection (red). The expected blue and red distributions are obtained by simulating the OU process 100 times for all the mammalian PLD sequences (*G*), and for the mammalian PLD sequences without primates (*H*). LR, likelihood ratio.

To understand whether these amino acid switches are caused by neutral evolution or positive selection, we estimated how strongly evolutionary rates vary across the amino acid sites within the PLD, and along the branches of its phylogenetic tree (see Materials and Methods for details) (Yang and Dos Reis 2011). We detected positive selection in 10 branches (*P* value < 0.05) and 12 sites (probability > 0.90) (fig. 2*A* andsupplementary table S3, Supplementary Material online) and in three types of substitutions: G to S, S to G, and Q to P. We observed the highest likelihood of positive selection for serine at the sites 42, 119, 129, and 131 and threonine at the sites 40 and 71 which occurred in the branches leading to primates and greater apes (supplementary information, Supplementary Material online).

The positively selected residues in primates (i.e., sites 42, 71, 78, 129, and 131) are among the sites in human FUS that are phosphorylated at serine and threonine (Monahan et al. 2017; Rhoads et al. 2018). Their phosphorylation not only increases the recruitment of FUS to the sites of DNA damage (Wang et al. 2013) but also inhibits the time-dependent changes in the material properties from liquid-like to solid-like states (Monahan et al. 2017). From a total of 32 phosphosites, only nine sites (i.e., sites 3, 7, 11, 26, 57, 77, 87, 96, and 148) were fully conserved, but the rest (24 sites) switched forth and back between only two pairs of amino acids (G-S and A-T) (fig. 2*B*). These sites occurred in both evolutionary hotspots, and their evolutionary rates were significantly higher than for the rest of the PLD residues (fig. 2*E*; Wilcoxon rank-sum test, *P* = 7.9 × 10^−4^, supplementary fig. S3, Supplementary Material online). The PLD sequences of primates and great apes harbor an exceptionally large number of phosphosites. That is, they harbor 29 and 31 phosphosites, respectively, which is 3 and 6 sites more than the average number of mammalian PLD phosphosites (fig. 2*E*). We found that the ratio of phosphosite-creating substitutions to phosphosite-destroying substitutions (i.e., [G to S + A to T]/[S to G + T to A]) is 0.67 in the whole phylogenetic tree, but significantly increases to 2.75 in the primate clade (Fisher’s exact test, *P* = 0.011; fig. 2*C*, 2*D* and supplementary information, Supplementary Material online). In other words, positively selected G to S and A to T substitutions have significantly increased the total number of phosphosites in the PLD sequences of primates.

Outside of the primates, the average number of phosphosites in the mammalian PLD sequences is ∼26 ± 2 sites. This small variation might indicate that the total number of S/T amino acids in these sites is stabilized in the evolution of the FUS PLD in mammals. To find out whether stabilizing selection has acted on our FUS sequences, we compared the likelihood that genetic drift alone or drift together with selection acted on the total number of phosphosites using an Ornstein–Uhlenbeck (OU) process (Butler and King 2004). This process has been used to compare the likelihood of drift alone with that of drift and selection in the evolution of different traits and characters (Hansen et al. 2000) (fig. 2*F*; see Materials and Methods for details). We found that stabilizing selection better explains the evolution of phosphosites than pure drift (likelihood ratio test, *P* = 0.015, fig. 2G;supplementary table S4, Supplementary Material online). We also asked whether two regimes (primate/nonprimate) of phosphosite evolution can better explain evolutionary rates than a single regime. We did so by comparing the likelihood of a two-optimum OU process given the data with that of a one-optimum OU process. The likelihood of the two-optimum process was significantly greater than the one-optimum process (supplementary table S4, Supplementary Material online; likelihood ratio test, *P* = 0.045). To ensure that the small size of one data set (22 species in the primate clade) did not bias our calculations, as has been observed in other applications of OU processes (Cooper et al. 2016), we further calculated the likelihood of stabilizing selection in the evolution of mammals without primates. The signature of stabilizing selection increases dramatically when primates are removed from this analysis (fig. 2*H*; likelihood ratio test, *P* = 2.28 × 10^−7^; supplementary table S4, Supplementary Material online). Together with our phylogenetic analysis of positive selection (fig. 2*A*), these observations suggest two regimes in the evolution of FUS phosphosites. In mammals except for primates, the number of phosphosites is under stabilizing selection. In primates and, in particular in great apes, positive selection has further increased the number of phosphorylation sites.

**Fig. 3.**
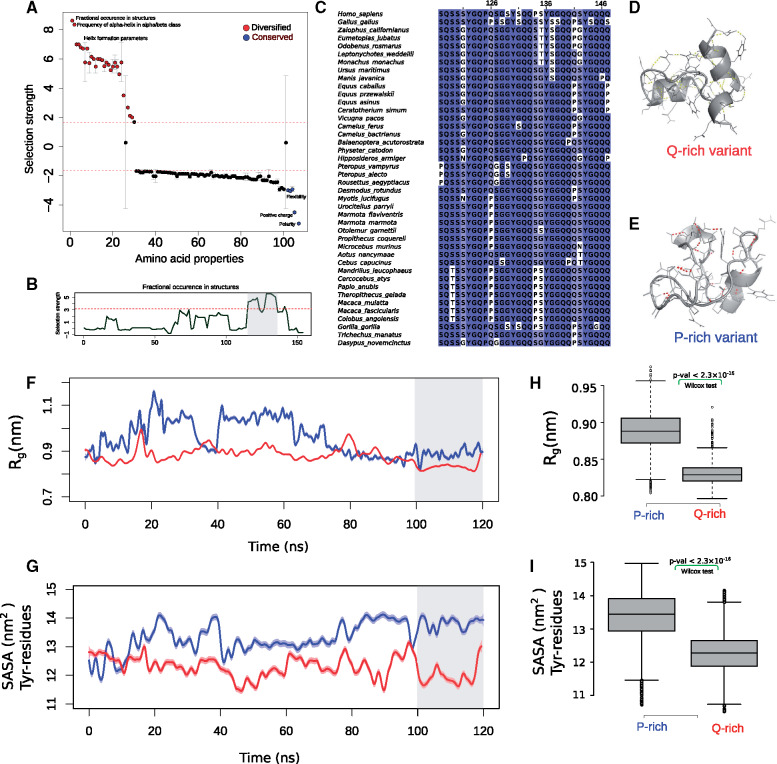
Proline substitutions disrupt the formation of secondary structure in an evolutionary PLD hotspot. (*A*) In total, 108 different amino acid properties showing radical changes in the evolution of the PLD, ranked by selection’s tendency to either help diversify them (positive values, red) or conserve them (negative values, blue) during PLD evolution. Properties falling above and below the dashed line are selected for or against at a high significance level of 0.001. Phosphosites are excluded from this analysis. Because several amino properties correlate with each other, we averaged the selection’s tendency between highly correlated properties (Spearman’s correlation *R* > 0.8). Error bars show one standard deviation of selection strength between highly correlated amino acid properties. Selection tends to diversify properties that change the structure-formation propensity, whereas it tends to conserve amino acid polarity and flexibility. (*B*) Selection strength of the property “fractional occurrence in tetrapeptides in protein structures (RACS820101; Rackovsky and Scheraga 1982)” over the length of the PLD (horizontal axis). The area highlighted in blue shows the region S117 to S147, where selection to diversify this property is maximal. (*C*) Multiple sequence alignment of the segment S117 to S147 for selected mammalian PLD sequences. Color saturation represents the frequency of the amino acid in each column, and ranges from dark blue (>80%) to white (<40%). (*D*) PEP-FOLD predicted structure of the glutamine-rich, and (*E*) the proline-rich variant of the region S117 to S147. The sites 126, 132, 140, 141, 146, and 147 were computationally substituted to glutamine or proline to create the Q-rich or the P-rich variants, respectively. Both structures were generated by the PEP-FOLD webserver (Maupetit et al. 2009). (*F*) The radius of gyration per simulation time for the P-rich (shown in blue) and the Q-rich (shown in red) variants. (*G*) The solvent accessible surface area (Eisenhaber et al. 1995) of tyrosine sticker residues per simulation time for the P-rich (shown in blue) and the Q-rich (shown in red) variants. (*H*) Boxplots comparing the radius of gyration of the P-rich and the Q-rich variants. (*I*) Boxplots comparing the solvent accessible area of tyrosine sticker residues. All *P* values were calculated using the Wilcoxon test.

The disordered domains of proteins, in particular proteins that undergo phase separation, preserve key amino acid features such as charge and sequence composition throughout their evolution (Zarin et al. 2017, 2019). We thus examined the physicochemical properties that are either conserved or positively selected in the evolution of the PLD in mammalian FUS (see Materials and Methods for details). We found that amino acid substitutions in the PLD have significantly conserved polarity, flexibility, and solvation free energy (fig. 3*A* and supplementary table S5, Supplementary Material online; chi-square goodness-of-fit, *P* < 10^−7^). We also found several properties whose changes were more frequent than expected from strict neutrality, and that had diversified in the evolution of the PLD (fig. 3*A*). The most significantly diversified property is the average occurrence of amino acids in a tetrapeptide unit in protein structures (Rackovsky and Scheraga, 1982). This property quantifies the nucleation propensity of amino acids in segments of four residues and divides the amino acids into two groups. Tetrapeptides with amino acids in the first group (Pro, Gly, His, Tyr, Cys, Asn, and Trp) are more likely to adopt extended structures. Tetrapeptides with amino acids in the second group (all other amino acids) are more likely to form helical and bend conformations.

For this property, amino acid substitutions in the region S117 to S147 had the maximum strength of positive selection (fig. 3*B* and supplementary table S6, Supplementary Material online). This region is enriched in substitutions involving proline (i.e., in residues 105, 117, 126, 132, 134, 140, 141, 146, and 147; fig. 3*C*). Importantly, our analysis of positive selection had shown that Q to P substitutions are positively selected in different branches of the phylogenetic tree (e.g., Q141P in Gorilla (*Gorilla gorilla*) and S134P in the branch leading to primates; supplementary information, Supplementary Material online).

To study the effect of proline substitutions in this evolutionary hotspot, we created in silico a glutamine-rich and a proline-rich variant from the region S117 to S147 by selecting Q and P in all residues that had experienced Q to P substitutions in different mammalian sequences (residues 126, 132, 140, 141, 146, and 147), respectively. We then predicted the secondary structure content of the two variants using the PEP-FOLD algorithm (Maupetit et al. 2009) and validated the stability of these predictions using molecular dynamics simulations (see Materials and Methods for details). The glutamine-rich variant forms three short helices that extend from residue Q118 to S121, from S129 to S135, and from Q139 to G144 (fig. 3*D*). In contrast, the proline-rich variant is mostly unstructured and retains only partially the middle of the three helices (Q126 to Y129; fig. 3*E*). We further performed molecular dynamics simulations for 120 ns on these initial structures. Importantly, the Q-rich variant showed higher helical content (supplementary fig. S6*A*, Supplementary Material online) and, on average, five more side-chain hydrogen bonds compared with the P-rich variant (supplementary fig. S6*B*, Supplementary Material online). The P-rich variant had an increased radius of gyration compared with the Q-rich variant (fig. 3*F* and *H*; Wilcoxon rank-sum test, *P* < 2.2 × 10^−16^), and its tyrosine residues were on average more exposed to solvent (fig. 3*F* and *I*; Wilcoxon rank-sum test, *P* < 2.2 × 10^−16^). We thus conclude that Q to P substitutions help maintain an unstructured state and increase the likelihood of the interactions of the sticker tyrosine residues with arginines in the RNA-binding domain of FUS.

**Fig. 4.**
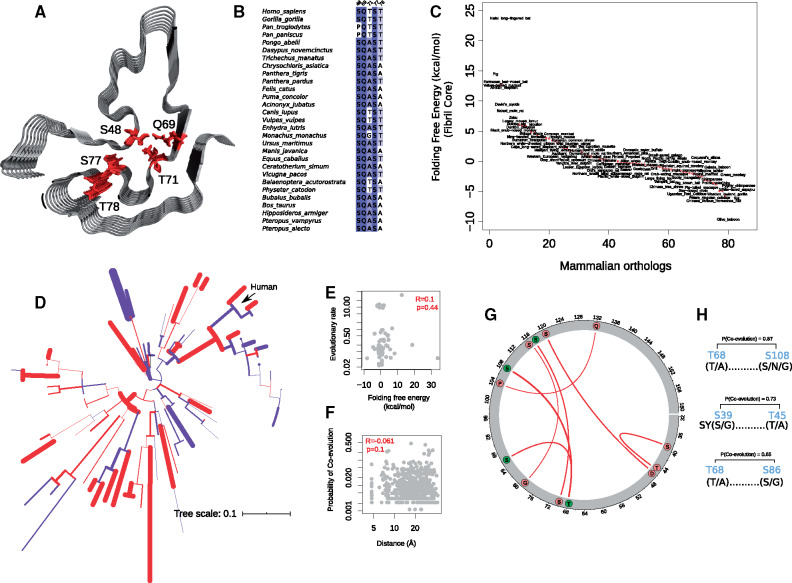
The stability of the fibril core varies substantially among mammalian PLD sequences. (*A*) Structure of the fibril core in the human PLD (PDB ID: 5W3N; Kapeli et al. 2016) with the residues S48, Q69, T71, S77, and T78 shown in red. (*B*) Sequence alignment of these residues for selected mammals. (*C*) Predicted free energy of folding of the fibril core, ranked for different mammalian PLDs. The stability of the human fibril core is taken as the reference free energy level of zero. (*D*) Mammalian phylogenetic tree showing branches with more stable (red) or less stable (blue) fibril cores compared with the structure of the human fibril core. (*E*) Evolutionary rate (d*N*/d*S*) of the PLD in the branches of the phylogenetic tree versus the change in the predicted folding free energy of the fibril core compared with the human fibril core. (*F*) Probability of coevolution between pairs of residues versus their distance on the PDB structure of the human fibril core (PDB ID: 5W3N; Kapeli et al. 2016). “R” indicates Spearman’s rank correlation coefficient. (*G*) Circos map showing PLD residue pairs that coevolve with high probability (red, *P* > 0.5). Residues phosphorylated in human FUS are shown as green circles. (*H*) High probability of coevolution observed in the pairs T68-S108, S39-T45, and T68-S86. For each residue, the most frequent amino acids are shown in parenthesis.

Finally, we examined the changes in the propensity of fibril formation in the evolution of the PLD in mammalian FUS. The first hotspot in the PLD, from S30 to S86, corresponds to a region that forms a fibrillar beta-sheet structure at high concentrations (fig. 4*A*) (Murray et al. 2017). The formation of these cross-beta sheet structures has been proposed (Murray et al. 2017) (but also disputed; Burke et al. 2015; Franzmann and Alberti 2019) to drive phase separation of FUS. Strikingly, we observed hydrogen-bond breaking substitutions in these regions that abolish side-chain hydrogen bonding and likely destabilize the fibril core (fig. 4*B*). For example, T78 and S84, which form inter-residue hydrogen bonds in the structure of the fibril core, are repeatedly substituted to alanine and glycine in different mammals. Other examples include alanine or proline substitutions in the residues S48, Q69, and T71, which hydrogen-bond and join the segment S44 to Y50 with T64 to G80. We further predicted the stability of fibril cores in different mammals and found a substantial variation in free energy of folding (fig. 4*C* and supplementary table S7, Supplementary Material online) which is more likely caused by pure drift than stabilizing selection (likelihood ratio test, *P* = 0.019; supplementary table S8, Supplementary Material online). In line with this observation, evolutionary rates of the PLD sequences were not significantly higher along the branches leading to fibrils with higher stabilities (fig. 4*D* and *E*; Spearman’s rank correlation, *R* = 0.047, *P* = 0.71).

We also pursued a complementary analysis to study the coevolution of fibril core residues. If the fibril core has remained intact during mammalian evolution, then we would expect that residues in the fibril core coevolve with each other, and possibly also with the rest of the PLD. To find out, we used Bayesian Graphical Models (Poon et al. 2008) to estimate the probability of coevolution between pairs of residues in the fibril core (see Materials and Methods). Despite the presence of pairs of coevolving residues (i.e., S39-T45, T68-S86, S68-S108, and G79-S116), we found no correlation between the probability of coevolution and the distance of coevolving residues on the 3D structure (fig. 4*F*; Spearman’s rank correlation, *R* = −0.061, *P* = 0.13). The coevolving residues T68 and S86 are phosphorylated in human FUS, showing that coevolution has modulated the phosphorylation potential of the PLD (fig. 4*G* and *H*). Altogether, our analyses reveal that the stability and structural integrity of the fibril core are not likely to be maintained during PLD evolution in mammalian FUS.

## Discussion

Our study reveals that the PLD is the most rapidly evolving domain of mammalian FUS. Within the PLD, the total number of phosphorylation sites has generally evolved under stabilizing selection and has been maintained at ∼26 sites per protein. We found that positive selection has significantly increased the number of phosphorylation sites in primates, particularly in great apes. Stabilizing selection has previously been proposed as a major process governing the evolution of phosphosites (Landry et al. 2009, 2014). According to this proposal, the cluster of phosphosites acts as a functional unit and is best described as a complex trait consisting of individual residues. Evolution then ensures that enough phosphorylation sites are retained for proper protein function (Landry et al. 2014). The evolution of mammalian FUS sequences provides a compelling case for this proposal and suggests that the total number of phosphosites can adapt to new regimes.

We also found that positive selection has favored glutamine to proline substitutions in an evolutionary hotspot of the PLD, likely to avoid secondary structure formation and maintain an unstructured state in the liquid state of FUS. Previously, proline substitutions had been shown to reduce the formation of irreversible aggregates of the huntingtin protein in Huntington disease (Popiel et al. 2004). Our analysis extends this observation to the much greater evolutionary time scale of mammalian evolution, which unfolded over 160 My.

The evolutionary pattern of the PLD in mammals supports the polymer-based “molecular grammar” of phase separation in FUS (Wang et al. 2018). Specifically, the complete conservation of the number as well as position of tyrosines is in line with the role of these residues as stickers in associative polymers. This observation further extends a recent observation that conserved patterning of aromatic residues is important for the phase-separation behavior of PLDs (Martin et al. 2020). Also, the strong conservation of polarity, flexibility, and solvation free energy in the regions that separate the tyrosine residues ensures that these regions can act as flexible spacers to keep the PLD chain flexible in the liquid state of FUS. Whether this conservation pattern in sticker number and patterning extends to other phase-separating proteins is an important question for future research.

We also examined the relevance of cross-beta sheet fibril formation in the evolution of the PLD. We did not observe selection to either maintain the stability of the fibril core or destabilize its structure, suggesting that fibril core stability has evolved under genetic drift alone. Consistent with this notion is our observation that the amino acids that contact each other in the fibril core of human FUS do not coevolve. Because naturally occurring substitutions disrupt the hydrogen bonding that is necessary for the stability of the fibril core, it is unlikely that the formation of this core is a universal driver of FUS self-assembly in mammals. However, our results do not reject the possibility that the fibril core forms, because we found fibril core structures in mammals that are more stable than the structure of the fibril core in human FUS. Also, we performed our computations on the 3D structure of human FUS, such that the absence of biochemical and structural data on nonhuman FUS orthologs might affect our results. To assess the importance of hydrogen-bond breaking substitutions on the structure of FUS more generally, it will be necessary to resolve the structure of nonhuman FUS orthologs with fibril cores that are less stable than the human fibril core, using methods such as solid-state nuclear magnetic resonance. Likewise, we cannot rule out that the PLD in different FUS orthologs forms amyloid polymorphs. Cryogenic transmission electron microscopy (cryo-EM) can help determine whether amyloid morphology varies among mammalian FUS orthologs. If fibril formation is not observed experimentally in FUS orthologs, we predict that phosphorylation or frequent core-destabilizing substitutions (mainly S to G and T to A) help avoid the formation of fibrils, which can mature into solid-like and irreversible aggregates.

Altogether, our work suggests that properties affecting phase separation may be evolvable in FUS, an essential protein that is also involved in neurological diseases. To prove this evolvability will require experimental studies. We propose the following experiments to validate our computational predictions. First and foremost, phase-separation assays with purified proteins can assess whether positively selected serine residues are functional and influence the time-dependent changes in the material states of FUS assemblies. For example, one can quantify the aggregation propensity of phosphomimetic FUS mutants in these residues (Monahan et al. 2017). One can also quantify and compare aggregation propensity of proline substitutions in positively selected proline residues among different FUS orthologs. Second, fluorescence recovery after photobleaching can help find out whether the hardening and softening of FUS droplets are substantially affected by substitutions between glycine and serine, alanine and threonine, and proline and glutamine (Wang et al. 2018). Third, small-angle X-ray scattering of the truncated evolutionary hotspot sequences, as well as hydrogen-deuterium exchange measurements of tyrosine residues in these segments can help determine whether evolution has altered the radii of gyration, as well as the accessibility of tyrosine residues for interaction with arginines in the RNA-binding domain of FUS.

The lack of protein structure in the PLD of FUS leads to substantial sequence variation, a feature that is common in intrinsically disordered proteins (Brown et al. 2002; Lin et al. 2007; Chen et al. 2011; Xue et al. 2013). Given this high overall divergence, stabilizing the condensed liquid-droplet state requires evolutionary mechanisms to maintain the disordered nature of the protein and avoid liquid-to-solid phase transitions. Our observations show that stabilizing selection of phosphorylation sites and positive selection of proline substitutions are two primary mechanisms to maintain the phase-separation propensity of FUS in mammals. We expect these mechanisms to hold for the evolution of the large set of ∼2,600 proteins in the human proteome with similar sequence architecture to FUS. These proteins, including the members of the FUS-like family of proteins such as EWS and TAF15, have prion-like and RNA-binding domains that are similar in length and composition to these domains in FUS, and might function as scaffolds for biomolecular condensates in the cell (Wang et al. 2018).

## Materials and Methods

### Data Compilation

We retrieved 105 coding sequences of mammalian FUS genes from the NCBI (Pruitt et al. 2007) and ENSEMBL (Hubbard 2002) databases. We subdivided these sequences into three subsets. We used the first of these subsets, which comprised 105 mammalian sequences, to build a multiple sequence alignment for the calculation of sequence entropy. We used the second subset of 85 sequences, which had confident phylogenetic support from the TimeTree database (Hedges et al. 2006) to 1) estimate the likelihood of drift and selection with the aid of an OU process, 2) correlate the evolutionary rates of the PLD with the stability of the fibril core’s structure, and 3) to analyze coevolution of the PLD residues. This sequence alignment was highly conserved and indels were infrequent. From 160 residues in 85 mammals, only 14 positions were deleted in less than 3 species. The average conservation per residue, calculated from the BLOSUM 62 substitution matrix, was 0.92 ± 0.13. Finally, we used a third subset of 50 sequences with diverse taxonomic sampling in our analysis of positive selection. (The accession numbers of all sequences used in this work, as well as the sequences of reconstructed ancestors are available in the supplementary information, Supplementary Material online.)

### Estimating Evolution Rate and Detecting Positive Evolution

We prepared protein sequence alignments with the codon-based CLUSTAL algorithm (Sievers et al. 2011) implemented in MEGA (Kumar et al. 2016) and Aliview (Larsson 2014) using the default parameters. We used the codeml program within the PAML suite (Yang 2007) to obtain maximum-likelihood estimates of the ratio d*N*/d*S*, that is, the ratio of the number of nonsynonymous substitutions per nonsynonymous site to the number of synonymous substitutions per synonymous site. This ratio is a widely used measure of selection strength on an evolving sequence (Yang 2006). For the estimation of d*N*/d*S*, we used the equilibrium codon frequencies from the products of the average observed frequencies in the three codon positions using the F3X4 model (Yang 2007). We tested the likelihood of positive selection in our sequences using the branch-site test for positive selection (Yang and Dos Reis 2011). In this model, a phylogenetic tree is partitioned into the foreground and background branches. The likelihoods of dN/dS > 1 and dN/dS = 1 along the foreground branches are compared using likelihood ratio tests. We determined the posterior probabilities that specific sites (amino acids) are subject to positive selection using the Bayes Empirical Bayes (Yang et al. 2005) method implemented in PAML (Yang 2007).

To mitigate the high rates of false positives in the detection of positively selected residues when indels are present, we performed two complementary sets of calculations. In the first set, we treated gaps as ambiguous data using the cleandata = 0 command in PAML. In the second set, we removed all positions that contained gap using the cleandata = 1 command. Overall, we only observed 6 out of 97 branches whose likelihood values were significantly affected by the removal of indels. Importantly, the likelihood of positive selection along the branches leading to primates was unaffected (supplementary table S10, Supplementary Material online).

### Ancestral Sequence Reconstruction

To reconstruct ancestral sequences, we fitted different substitution models to our data (PLD sequences and the mammalian phylogenetic tree), allowing that evolutionary rates may vary among protein sites. The substitution model JTT (Jones et al. 1992) with the gamma distribution of evolutionary rates had the highest Bayesian Information Criterion score (Burke et al. 2015). We thus used this model and inferred ancestral PLD sequences using the maximum-likelihood method implemented in MEGA7 (Kumar et al. 2016). The probabilities of the most likely inferred amino acids were on average ∼0.93 ± 0.16. We also reconstructed ancestors using a substitution matrix developed to study the evolution of intrinsically disordered mammalian proteins (Szalkowski and Anisimova 2011). For this matrix too, the probabilities of reconstructed ancestral sequences were ∼ 0.92 ± 0.19.

### Detection of Amino Acid Properties under Selection

We used the TreeSAAP method (Woolley et al. 2003) to infer how natural selection may change amino acid properties in the evolution of the PLD. Briefly, this method compares the distribution of changes in amino acid properties along the branches of a phylogenetic tree with an expected distribution, using the codon composition of a set of extant sequences. Changes in amino acid properties are divided into eight categories, from the most conserved (category 1) to the most radical changes (category 8). The method then calculates the goodness-of-fit (χ^2^-distribution) between the expected and the observed frequencies and tests the hypothesis that these distributions are equal for each amino acid property. For a specific property, the deviation between observed and expected frequencies in each category is calculated using a Z-score. We refer to this Z-score as the deviation from neutrality or the selection strength throughout this paper. A highly significant Z-score (Z > 3.09, *P* < 0.01) shows that more nonsynonymous substitutions change the property of interest than expected from neutral evolution. Although TreeSAAP was originally devised to study the evolution of structured proteins, the conserved sequence alignment of the PLD in mammals justifies the use of this approach in this work. The limited usage of amino acids in low complexity regions renders the expected distribution of properties dramatically different between structured and unstructured proteins. Nonetheless, if the expected distribution is built from the extant sequences, the inference of selection should be robust.

### Detection of Coevolution

To infer the coevolutionary history of protein sites within the PLD, we used Bayesian Graphical Models (Poon et al. 2008) implemented in the HyPhy package (Pond and Muse 2005). We first used our ancestral reconstruction of FUS to construct a binary matrix representing the presence and absence of substitutions on each branch (rows) of the phylogenetic tree and in each site of the protein (columns). The joint distribution of all substitutions was then inferred using Bayesian networks and Markov Chain Monte Carlo sampling with default parameters in the SpiderMonkey method (Poon et al. 2008). We avoided the use of mutual information to infer coevolution because it leads to a high rate of false positives in the detection of coevolving sites when sequences are substantially similar (Harmon et al. 2017) (∼>62%), as in our case.

### Prediction of Folding Free Energy

We predicted the stability of the structure of the fibril core of the PLD in different mammals. We generated the 3D structures of the fibril core using its structure in human (PDB ID: 5W3N; Murray et al. 2017) as a template. We then calculated the free energy of folding of the fibril core made from the mammalian PLD sequences using the FoldX algorithm, which uses an empirical force field for the prediction of the free energy change of protein structures upon mutations (Schymkowitz et al. 2005; Buß et al. 2018). We first minimized the free energy of this structure using the Repair command in FoldX (Schymkowitz et al. 2005). We then created in silico mutants of this structure to create different mammalian PLD orthologs using the BuildModel command of FoldX (Schymkowitz et al. 2005).

### OU Processes

To estimate the significance of stabilizing selection versus pure drift, we used OU processes that are corrected for phylogenetic dependence of species (Hansen et al. 2000). These models have been used to test various evolutionary hypotheses in the evolution of different characters and traits (Butler and King 2004), gene expression level (Rohlfs et al. 2014), and protein structure (Challis and Schmidler 2012). In brief, these models assume that the character of interest, *X*(*t*), evolves in time unit (*t*), according to an OU process:

(1) $$dX_{i}\left(t\right)=\alpha \left[\theta -X_{i}\left(t\right)\right]dt+\sigma dB_{i}\left(t\right)$$

The parameter θ is the optimum value of *X*(*t*) in the *i*th lineage and the parameters α and σ represent the strength of selectin and drift, respectively. The term dB_*i*_(*t*) is a white noise term, with mean 0 and variance d*t*. Equation (1) defines a Gaussian process whose moments depend on the parameters θ, α, and σ and the total time spent for a character in the lineage of interest, *t* = *T*. *T* is either defined as the evolutionary time or equal to the branch length, as in our case. The values of the optimum parameter of the evolving trait (θ) can vary according to the assumed evolutionary process. For example, if the character of interest evolves under a single optimum, θ is the same for all lineages. These parameters are estimated by minimizing the logarithm of a likelihood function that assumes multivariate normality of all characters at the terminal taxa, *X*(*t* = *T*).

We used the total number of phosphosites and the folding free energy as the traits of interest in evolution. We then fitted the models for pure drift and stabilizing selection using the BROWN and HANSEN commands in the OUCH package (Butler and King 2004), respectively. The input to these commands was the mammalian phylogenetic tree in Newick format, together with a data vector of the trait of interest, either the total number of phosphosites or the stability of the fibril core. We used the initial values of α=1 and σ=1 to initialize the optimization process using the Nelder and Mead simplex algorithm (Nelder and Mead 1965). For modeling stabilizing selection, we assumed that all nodes belong to a single selective regime. We used likelihood ratio tests to compare the likelihoods of drift and selection.

### Prediction of Peptide Structure and Molecular Dynamics Simulations

We generated the initial structures of the glutamine-rich and the proline-rich variants using the PEP-FOLD online web server (Maupetit et al. 2009). For molecular dynamics simulations, we used the GROMACS package (Van Der Spoel et al. 2005) (v2019a) and employed periodic boundary conditions at 300 K and 1 atm, with a time step of 2 fs. We chose the Gromos 54a7 (Schmid et al. 2011) force field because of its ability to reproduce the kinetics of helix formation (Damas et al. 2013). We kept the temperature and pressure constant with the Nose–Hoover thermostat (Nosé 1984; Hoover 1986) (time constant = 0.1 ps) and the Parinello–Rahman barostat (Parrinello and Rahman 1980) (time constant = 1.5 ps), respectively. For both van der Waals and short-ranged Coulombic interactions, we used a cutoff radius of 1.0 nm and used the particle-particle mesh Ewald method for the long-ranged Coulombic interactions (Van Der Spoel et al. 2005). We minimized the energy of both structures by the steepest descent method, followed by a position-restraint simulation to equilibrate the water molecules. We then performed a grand canonical ensemble simulation (constant number of particles, temperature, and pressure) at 300 K for 120 ns and calculated the radii of gyration, solvent accessible surface area of tyrosine residues, percentage helicity of different residues, and the number of side-chain hydrogen bonds. We performed all statistical analyses in R, using scripts available on GitHub (https://github.com/dasmeh/FUSEVOL).

## Supplementary Material

Supplementary data are available at *Molecular Biology and Evolution* online.

## Data Availability

All data generated or analyzed during this study are available in the repository: https://github.com/dasmeh/FUSEVOL

## Supplementary Material

msaa258_Supplementary_Data
