# Supplementary material for: Natural Selection on the Phase-Separation Properties of FUS during 160 My of Mammalian Evolution
Source: Mol Biol Evol. 2020 Oct 6;38(3):940–51. doi: 10.1093/molbev/msaa258 (PMC7947763; doi:10.1093/molbev/msaa258)
Supplement: msaa258_Supplementary_Data [file msaa258_supplementary_data.zip › Supplementary_Info_MBE.docx]

**Supplementary Information**

**Natural selection on the phase-separation properties of FUS during 160 million years of mammalian evolution**

Pouria Dasmeh^1,2^ & Andreas Wagner^1^

^1^Institute for Evolutionary Biology and Environmental Studies, University of Zurich, Zurich, Switzerland. ^2^Department of Chemistry and Chemical Biology, Harvard University, Cambridge, MA USA 02139.

1. **Supplementary Figures:**

**Figure S1.** A) The criteria for positive selection (Twice the difference in the logarithm of likelihood function) for different branches of the phylogenetic tree. The branches leading to species in the orders Artiodactyla and primates have significant probability of positive selection. B) The probability of per-branch selection of amino acid sites (i.e., the branch probability multiplied by the site probability) for different branches of the phylogenetic tree.

**Figure S2.** Substitution patterns mapped on the phylogenetic tree of mammals in three representative sites under positive selection, A) S147, B) G56, and C) 148. See the supplementary results for the list of all transitions under positive selection.


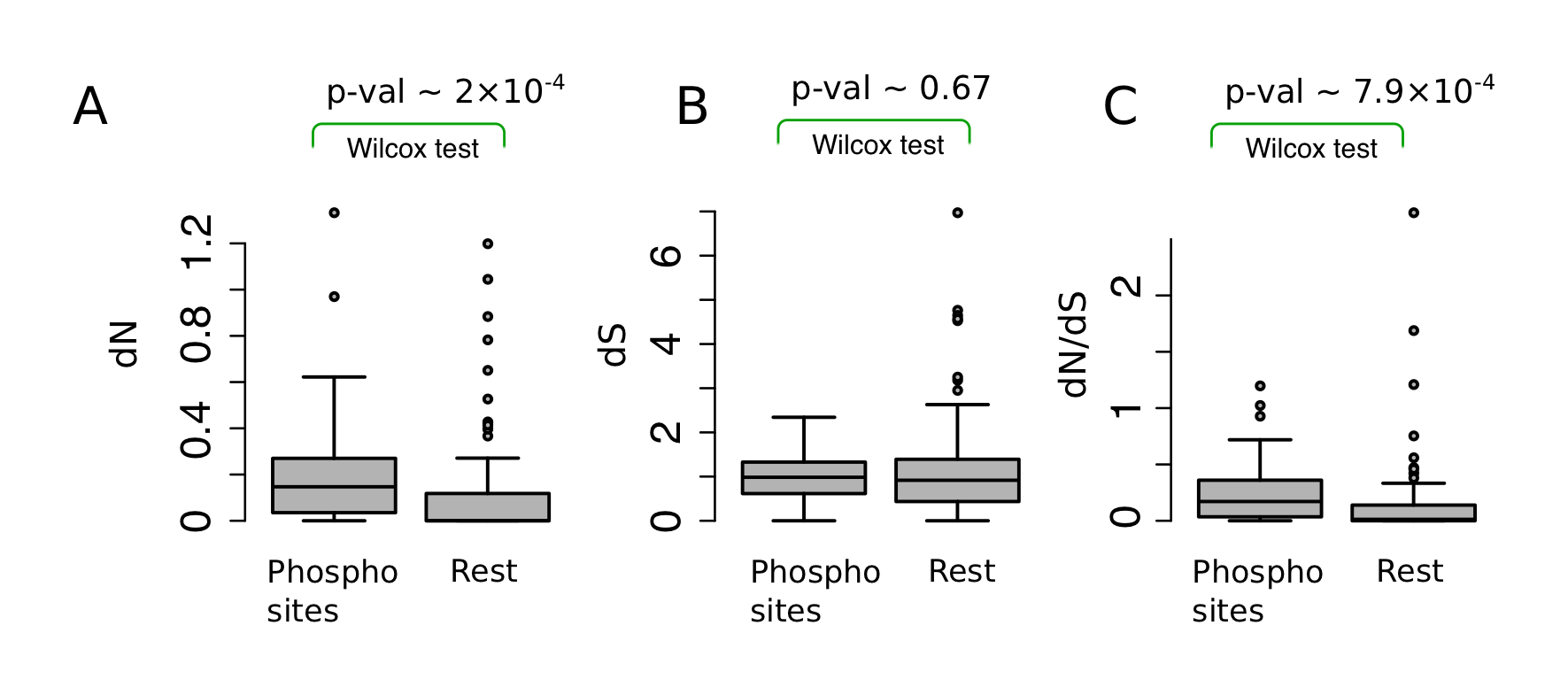


**Figure S3.** The rate of nonsynonymous substitutions, d*N* (panel A), Synonymous substitutions, d*S* (panel B), and evolutionary rate, d*N*/d*S* (panel C), for phosphorylates sites and the rest of the PLD residues.

**Figure S4.** Ancestral state mapping in the PLD for sites with the reconstruction probability > 0.9. Each circle or node represents one amino acid, and substitutions are shown as edges that connect these nodes. The thickness of each edge corresponds to the number of substitutions between the two incident nodes. Ancestral sequences are reconstructed with the JTT substitution matrix.

**Figure S5.** Ancestral state mapping in the PLD for sites with the reconstruction probability > 0.9. Each circle or node represents one amino acid, and substitutions are shown as edges that connect these nodes. The thickness of each edge corresponds to the number of substitutions between the two incident nodes. Ancestral sequences are reconstructed with the substitution matrix built from intrinsically disordered proteins in mammals^1^.

**Figure S6.** A) The percentage of simulation time that any one residue (horizontal axis) is found in a helical conformation and B) the number of side-chain hydrogen bonds in the molecular dynamics simulations of the Q-rich (red) and the P-rich (blue) variants. The p-value was calculated with Wilcoxon rank-sum test.

1. **Supplementary Tables:**

- Table S1: The substitution patterns of the ancestrlal reconstruction in the PLD of FUS
- Table S2: The substitution patterns of the ancestrlal reconstruction in the PLD of FUS
- Table S3: The likelihoods for the alternative and the null model of branch-site test for positive selection
- Table S4: Ornstein-Uhlenbeck processes for phosphorylated sites (without primates)
- Table S5: Highly conserved and highly diversified physicochemical properties in the evolution of the PLD in FUS.
- Table S6: Z-score values of the property RACS820112 per sliding window of 5 codons.
- Table S7: FoldX free energy terms for extant and ancestral PLD sequences of mammalian FUS.
- Table S8: Ornstein-Uhlenbeck process for changes of the folding free energy in the evolution of fibril core in FUS.
- Table S9: Ancestral substitutions in the evolution of the PLD for primates and all mammals (sheet 1), and for all mammals calculated from the JTT matrix and from the IDR matrix (sheet2).
- Table S10: Branch-site test for positive selection with and without gaps in the alignment of the PLD in mammals.

1. **Supplementary files:**

All data are available at: <https://github.com/dasmeh/FUSEVOL>

- Sequences of mammalian FUS and ancestral sequences.
- Structures of Q-rich and P-rich variants made by PEP-fold algorithm.
- Structure of fibril cores in mammals made by FoldX *in silico* mutagenesis.

**Positive selection patterns along the branches**

**1)** Branch Number: 4

(Site, Probability, Transition)

64, 0.674, T > S

**2)** Branch Number: 19

(Site, Probability, Transition)

56, 0.776, S>G

148, 0.955, S>Q

Lineage in the phylogeny:

((((((((((((Bos_mutus_wild_yak:0.019457,Bos_indicus_zebu_cattle:0.006900):0.006263,Bos_taurus_cow:0.013914):0.070474,(Pantholops_hodgsonii_chiru:0.018526,(Capra_hircus_goat:0.000004,Ovis_aries_sheep:0.006562):0.014510):0.045189):0.147414,(Lipotes_vexillifer_Yangtze_River_dolphin:0.025717,Orcinus_orca_killer_whale:0.027657):0.054548):0.026334,Sus_scrofa_pig:0.110769):0.000969,Camelus_ferus_Wild_Bactrian_camel:0.169220):0.089605 🡨

**3)** Branch Number: 32

(Site, Probability, Transition)

56, 0.728, S>G

83, 0.617, S>G

Lineage in the phylogeny:

(((Ursus_maritimus_polar_bear:0.043702, Ailuropoda_melanoleuca_giant_panda:0.009459):0.034020 🡨

**4)** Branch Number: 33

(Site, Probability, Transition)

135, 0.690, G>T

Lineage in the phylogeny:

Odobenus_rosmarus_divergens_Pacific_walrus:0.050105 🡨

**5)** Branch Number: 46

(Site, Probability, Transition)

42, 0.819, S>G

56, 0.604, S>G

Lineage in the phylogeny:

((((((((Bos_mutus_wild_yak:0.019457,Bos_indicus_zebu_cattle:0.006900):0.006263,Bos_taurus_cow:0.013914):0.070474,(Pantholops_hodgsonii_chiru:0.018526,(Capra_hircus_goat:0.000004,Ovis_aries_sheep:0.006562):0.014510):0.045189):0.147414,(Lipotes_vexillifer_Yangtze_River_dolphin:0.025717,Orcinus_orca_killer_whale:0.027657):0.054548):0.026334,Sus_scrofa_pig:0.110769):0.000969,Camelus_ferus_Wild_Bactrian_camel:0.169220):0.089605,(((Equus_asinus_ass:0.006650,Equus_caballus_horse:0.000004):0.000004,Equus_przewalskii_Przewalskis_horse:0.006681):0.122260,((Panthera_tigris_altaica_Amur_tiger:0.000004,(Acinonyx_jubatus_cheetah:0.006612,Felis_catus_domestic_cat:0.013246):0.000004):0.052309,(((Ursus_maritimus_polar_bear:0.043702,Ailuropoda_melanoleuca_giant_panda:0.009459):0.034020,Odobenus_rosmarus_divergens_Pacific_walrus:0.050105):0.016635,Canis_familiaris_dog:0.040800):0.021089):0.042717):0.000004):0.012372,((Myotis_brandtii_Brandts_bat:0.071429,Myotis_davidii_Vesper_bat:0.034392):0.211993,(Hipposideros_armiger_great_roundleaf_bat:0.227675,Pteropus_alecto_black_fruit_bat:0.067987):0.044656):0.044726 🡨)

**6)** Branch Number: 61

(Site, Probability, Transition)

112, 0.598, S>G

(Tupaia_chinensis_Chinese_tree_shrew:0.168529 🡨)

**7)** Branch Number: 74

(Site, Probability, Transition)

40, 0.998, G>T

Lineage in the phylogeny:

(Pongo_abelii_Sumatran_orangutan:0.021484 🡨)

**8)** Branch Number: 75

(Site, Probability, Transition)

141, 0.761, Q>P

Lineage in the phylogeny:

(Gorilla_gorilla_gorilla_western_lowland_gorilla:0.021599 🡨)

**9)** Branch Number: 84

(Site, Probability, Transition)

42, 0.795, G>S

61, 0.823, T>S

119, 0.863, S>T

134, 0.875, S>P

148, 0.953, S>Q

149, 0.996, S>S

Lineage in the phylogeny:

(Callithrix_jacchus_whitetuftedear_marmoset:0.090719,((((Macaca_fascicularis_crabeating_macaque:0.006578,Macaca_mulatta_rhesus_monkey:0.000004):0.013118,Chlorocebus_sabaeus_green_monkey:0.019924):0.013720,(Rhinopithecus_bieti_black_snubnosed_monkey:0.000004,Rhinopithecus_roxellana_golden_snubnosed_monkey:0.000004):0.025800):0.006735,(Nomascus_leucogenys_northern_whitecheeked_gibbon:0.051824,(Pongo_abelii_Sumatran_orangutan:0.021484,(Gorilla_gorilla_gorilla_western_lowland_gorilla:0.021599,(Homo_sapiens_human:0.000004,(Pan_paniscus_bonobo:0.000004,Pan_troglodytes_chimpanzee:0.000004):0.013099):0.000004):0.041490):0.000004):0.017607):0.136167 🡨)

**10)** Branch Number: 88

(Site, Probability, Transition)

43, 0.974, Q>P

78, 0.592, T>G

82, 0.697, G>S

103, 0.973, Q>P

Lineage in the phylogeny:

Loxodonta_africana_African_savanna_elephant:0.120966 🡨

**Predicted evolutionary rates for each branch of the phylogenetic tree**

(branch lengths are the dN/dS values predicted by CODEML)

((((((((((((((Zalophus_californianus #999.0000 , Eumetopias_jubatus #244.3665 ) #225.7150 , Odobenus_rosmarus #689.4730 ) #397.1745 , (Leptonychotes_weddellii #0.0001 , Monachus_monachus #949.6424 ) #999.0000 ) #0.1697 , Enhydra_lutris #0.0001 ) #91.0856 , Ursus_maritimus #3.2462 ) #0.0001 , (Canis_lupus #999.0000 , Vulpes_vulpes #0.5161 ) #0.2592 ) #51.8415 , ((Panthera_tigris #116.6303 , Panthera_pardus #999.0000 ) #263.3471 , (Acinonyx_jubatus #638.3701 , (Felis_catus #0.0001 , Puma_concolor #293.8635 ) #336.2687 ) #271.5981 ) #0.2566 ) #0.0001 , Manis_javanica #0.2341 ) #0.0001 , (Ceratotherium_simum #0.2404 , (Equus_asinus #0.0001 , (Equus_caballus #195.2510 , Equus_przewalskii #238.5228 ) #142.0174 ) #0.2435 ) #0.0001 ) #70.8301 , (((Camelus_ferus #114.4290 , Camelus_bactrianus #116.8703 ) #0.2461 , Vicugna_pacos #0.0001 ) #0.2249 , (Sus_scrofa #0.3767 , ((((((Lagenorhynchus_obliquidens #0.0001 , Orcinus_orca #999.0000 ) #0.0001 , Delphinapterus_leucas #999.0000 ) #103.3377 , Lipotes_vexillifer #0.5114 ) #0.0001 , Physeter_catodon #0.2514 ) #999.0000 , Balaenoptera_acutorostrata #999.0000 ) #0.6677 , (((Bos_taurus #999.0000 , Bison_bison #198.5425 ) #0.7618 , Bubalus_bubalis #0.9989 ) #999.0000 , (Pantholops_hodgsonii #63.5889 , (Capra_hircus #171.4609 , Ovis_aries #0.0001 ) #0.0001 ) #0.3264 ) #0.0001 ) #0.0001 ) #0.0001 ) #0.2646 ) #147.3301 , (((Myotis_lucifugus #0.0001 , Miniopterus_natalensis #0.0001 ) #144.6956 , Desmodus_rotundus #0.7531 ) #1.3792 , (Hipposideros_armiger #0.1710 , (Rousettus_aegyptiacus #0.5025 , (Pteropus_vampyrus #0.0001 , Pteropus_alecto #71.8168 ) #0.0001 ) #0.1144 ) #27.6949 ) #1.1259 ) #0.0001 , (Erinaceus_europaeus #0.0958 , Condylura_cristata #0.2450 ) #1.2957 ) #0.0001 , (((((((((Rattus_norvegicus #0.1228 , Mus_musculus #0.0001 ) #0.0001 , Meriones_unguiculatus #0.0001 ) #0.0001 , (Peromyscus_maniculatus #0.0001 , (Mesocricetus_auratus #0.0001 , Cricetulus_griseus #0.0001 ) #0.0001 ) #135.4654 ) #0.0001 , Nannospalax_galili #0.2119 ) #0.0001 , Dipodomys_ordii #0.0001 ) #0.0001 , (Urocitellus_parryii #183.2193 , (Marmota_flaviventris #143.7105 , Marmota_marmota #0.0001 ) #0.0001 ) #0.1417 ) #0.0001 , (Heterocephalus_glaber #0.2017 , (Cavia_porcellus #0.6168 , Octodon_degus #0.2267 ) #0.0001 ) #0.0001 ) #0.0001 , (Oryctolagus_cuniculus #0.1365 , Ochotona_princeps #0.0358 ) #0.0001 ) #0.0001 , (((Propithecus_coquereli #999.0000 , Microcebus_murinus #0.0001 ) #0.0001 , Otolemur_garnettii #0.0001 ) #999.0000 , ((Aotus_nancymaae #0.3867 , Cebus_capucinus #0.0001 ) #0.0001 , (((((Pan_troglodytes #0.8493 , Pan_paniscus #146.8477 ) #999.0000 , Homo_sapiens #78.6724 ) #103.4384 , Gorilla_gorilla #77.7181 ) #0.2426 , Pongo_abelii #999.0000 ) #0.0001 , (((Rhinopithecus_roxellana #128.4902 , Rhinopithecus_bieti #0.9253 ) #0.4929 , (Piliocolobus_tephrosceles #0.2836 , Colobus_angolensis #95.7057 ) #102.5470 ) #87.3284 , (Chlorocebus_sabaeus #0.0001 , (((Macaca_mulatta #162.9342 , Macaca_fascicularis #153.6423 ) #145.0405 , Macaca_nemestrina #106.9026 ) #0.9058 , ((Mandrillus_leucophaeus #73.4895 , Cercocebus_atys #0.2822 ) #157.5496 , (Papio_anubis #0.2842 , Theropithecus_gelada #122.4933 ) #161.9117 ) #89.8523 ) #999.0000 ) #0.0001 ) #0.0001 ) #0.1470 ) #0.0001 ) #774.0332 ) #0.0001 ) #0.0001 , (Dasypus_novemcinctus #0.2869 , (Trichechus_manatus #0.3885 , (Chrysochloris_asiatica #0.0889 , Elephantulus_edwardii #0.0995 ) #0.4475 ) #0.0001 ) #0.0001 ) #0.0008 , Gallus_gallus #0.0038 );

**Sequences of Q-rich and P-rich variants**

>Q-rich

SQSSSYGQPQSGSYSQQSSYGGQQQSYGQQQ

>P-rich

PQSSSYGQPPSGSYSPQPSYGGQPPSYGQPP

**Fisher’s test for the significance of increased rate of phosphosite creating mutations in the primate clade**

|  | Primates | Rest of mammals |
| --- | --- | --- |
| G>S + A>T | 11 | 47 |
| S>G + T>A | 4 | 79 |

p.value calculated from Fisher’s exact test: 0.011

Sample estimates (odds ratio): 4.57

95% confidence interval: 1.26, 20.8

**Supplementary References**

1. Szalkowski, A.M. & Anisimova, M. Markov models of amino acid substitution to study proteins with intrinsically disordered regions. *PloS one* **6**, e20488 (2011).
